# A Web-based and Grid-enabled dChip version for the analysis of large sets of gene expression data

**DOI:** 10.1186/1471-2105-9-480

**Published:** 2008-11-13

**Authors:** Luca Corradi, Marco Fato, Ivan Porro, Silvia Scaglione, Livia Torterolo

**Affiliations:** 1 Computer Science, Systems, and Communication Department, University of Genova, Viale Causa 12, 16100 Genova, Italy

## Abstract

**Background:**

Microarray techniques are one of the main methods used to investigate thousands of gene expression profiles for enlightening complex biological processes responsible for serious diseases, with a great scientific impact and a wide application area. Several standalone applications had been developed in order to analyze microarray data. Two of the most known free analysis software packages are the R-based Bioconductor and dChip. The part of dChip software concerning the calculation and the analysis of gene expression has been modified to permit its execution on both cluster environments (supercomputers) and Grid infrastructures (distributed computing).

This work is not aimed at replacing existing tools, but it provides researchers with a method to analyze large datasets without any hardware or software constraints.

**Results:**

An application able to perform the computation and the analysis of gene expression on large datasets has been developed using algorithms provided by dChip. Different tests have been carried out in order to validate the results and to compare the performances obtained on different infrastructures. Validation tests have been performed using a small dataset related to the comparison of HUVEC (Human Umbilical Vein Endothelial Cells) and Fibroblasts, derived from same donors, treated with IFN-α.

Moreover performance tests have been executed just to compare performances on different environments using a large dataset including about 1000 samples related to Breast Cancer patients.

**Conclusion:**

A Grid-enabled software application for the analysis of large Microarray datasets has been proposed. DChip software has been ported on Linux platform and modified, using appropriate parallelization strategies, to permit its execution on both cluster environments and Grid infrastructures. The added value provided by the use of Grid technologies is the possibility to exploit both computational and data Grid infrastructures to analyze large datasets of distributed data. The software has been validated and performances on cluster and Grid environments have been compared obtaining quite good scalability results.

## Background

During the last years, genomics and proteomics have deeply changed the scientific approach to the study of the molecular basis of cells and tissues behaviors both in physiological and pathological conditions, giving a new comprehensive view to the research community.

As the interest on these fields has been more and more increasing, innovative and more suitable technologies have been developed. At present, one of the most promising and reactive fields is certainly the microarray technology, which has had, so far, a great scientific impact and a wide application area. In fact, several types of micorarrays have been developed and proposed, each focused on a specific type of analysis, from genetic screening to proteomics and from biological research to diagnostics.

Through the comparison of genomic profiles it is possible to study gene expression differences among cross-correlated conditions, thus understanding their meaning. Thanks to the microarray technology a large number of genes may be investigated at the same time to find which are differentially expressed on a certain cell type. Quantitative researchers have proposed a variety of methods for handling probe-level data from Affymetrix^® ^oligonucleotide arrays. Such methods employ different procedures for adjusting background fluorescence, normalizing data, incorporating information from "mismatch" probes, and summarizing probe sets.

Even if microarrays are a powerful instrument, studies on these data are often conditioned by technological limits, thus decreasing their capabilities. The most relevant limitation concerns the analysis of large datasets. In fact this kind of analysis requires long computational times rather than the availability of specific hardware. A huge availability of memory and computational power is required for analyzing microarrays and often researchers cannot succeed in performing their studies because of the impossibility to access suitable resources.

Several tools and algorithms had been developed in order to analyze microarray data, all of them consisting in standalone applications. Two of the most known free analysis software packages are the R-based Bioconductor and dChip [1,2].

This work is not aimed at replacing those systems, but it provides researchers with a new method to analyze large datasets without any hardware or software constraints, by simply using a common web browser. To reach this aim, dChip software has been modified by using appropriate parallelization strategies, to permit its execution on both cluster environments and Grid infrastructures, exploiting existing computational and storage capabilities. Since dChip is a wide application containing a large number of functionalities, this work is related to the computation and the analysis of gene expression.

## Implementation

The goal of this work is focused on the design and development of a tool for the analysis of gene expression to be included in a more general Grid-enabled software application for the analysis of microarray data. As an added value, the use of Grid technologies makes it possible to exploit both computational and data Grid infrastructures to analyze large datasets of distributed data.

DChip, an existing analysis tool for Microarray experiments, was originally a free and open source Windows application. Starting from the original source code (dChip 2005 version), several versions of the software have been implemented, in order to fit different kinds of resources [see Additional file 1]:

• standalone Linux version

• parallel cluster implementation

• parallel Grid implementation

The execution is supported on 64 bit computers too.

With regard to the user interface, as a first release, the application was implemented in a command line version to permit the execution on remote computing elements. Two input files are used: the first one contains specific options for the execution; the second one contains the list of the microarray files used for the analysis. As a second step, in order to simplify the use of above mentioned dChip versions, the executables have been integrated within a biomedical portal [3,4] which provides a simple graphical user interface to run the application. Such a portal integration allows unpractised users to store their experimental data on a complex storage system and access distributed data and services in a transparent way. Furthermore users can easily run the application from any computer or location with only Internet connection, without loosing time in installation and maintenance procedures. Moreover, users can use the software through a simple web interface and launch their analyses taking advantage of the possibility to orchestrate different portal services in a workflow strategy. Thanks to the ease of the web interface, users are not required to know technical dChip details.

**Figure 1:**
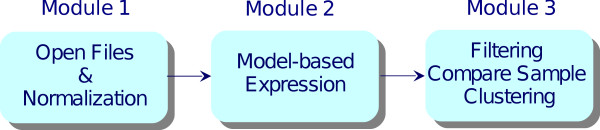
**Organization of dChip in different modules**. A graphical representation of developed dChip modules is shown. The original software was divided into three different modules concerning respectively (i) normalization, (ii) expression values computation, (iii) filtering, differentially expressed genes extraction and clustering.

The new software version has been designed to be modular, i.e. the original software has been divided into several independent modules, each performing a different part of the analysis. This approach has allowed to improve (i) optimization, by implementing the most appropriate parallelization strategy for each part of the analysis and (ii) scalability, by replacing in a transparent way one or more modules with other, more powerful, ones or with modules providing different functionalities. The application has been structured in three different modules that have to be executed sequentially (Figure 1):

• module 1: opening, reading and normalization of CEL files

• module 2: computation of expression levels

• module 3: filtering, extraction and clustering of differentially expressed genes

Each of them has been designed as a standalone program working in an independent way. Data and overall information are moved through the modules using CSV (Comma Separated Values) file format. The final output is composed of three main files containing respectively: the expression values in an R compatible format, the list of the differentially expressed genes and the cluster tree.

Using large datasets, long execution times and great computational efforts are required. Parallelization strategies are necessary to improve performances and to allow the analysis of a large number of arrays in a short time. A first accurate analysis of dChip algorithm revealed the possibility to parallelize both the first and the second module that implement the most data intensive algorithms from a computational point of view. The applied parallelization does not affect the original algorithms of dChip but it is related to a data access strategy. Since the algorithms for normalization and expression calculation work in different ways, two different parallelization approaches have been adopted.

The normalization algorithm is based on the invariant set method. It works by processing each array separately with a baseline chosen as the median intensity array. Therefore the Module 1 has been parallelized according to the number of microarrays. Each parallel execution opens a restricted number of files, normalizes them against the baseline and writes the related CSV output files.

DChip algorithms concerning the calculation of gene expression (PM only and PM/MM methods) work in a different way, since they need all genes from all microarrays to work. So the Module 2 has been parallelized into a number of executions, each reading all CVS files of all normalized arrays but performing the calculation only on a restricted group of genes. The execution results of each subset are then merged and the output thus containing the expression levels of all data is produced.

The third module reads the CSV file containing the gene expression values and allows to perform the filtering over genes, the extraction of differentially expressed genes and some clustering operations by using the dChip unmodified algorithms.

Two different modalities of parallel execution are available: with or without MPI (Message Passing Interface) libraries. The second approach allows the execution on environments not supporting MPI technology, but requires specific scripts for the management, the submission and the monitoring of parallel jobs.

Finally the code has been modified to enable the submission to the Grid infrastructure. For this purpose the gLite [5] middleware has been considered. In order to allow to read and to write files on remote and distributed storage elements, GFAL API [6] has been used. In this way it is possible to access data, reading the whole files, or a part of them, directly where they are stored without moving them to Grid elements that actually run the calculation. Thanks to a Public Key Infrastructure (PKI) [7], which provides X.509 certificate based authentications, this solution allows to preserve user privacy and data security.

## Results and discussion

An application able to perform the computation and the analysis of gene expression on large datasets of microarrays has been developed using dChip algorithms. In details, concerning pre-analysis, the invariant-set method has been used for normalization and PM-MM difference model or PM-only models can be chosen for genes expression calculation. Original dChip functionalities like filtering, differentially expressed genes (compare samples) discovery and clustering are provided by Module 3. Customized analyses can be performed by setting specific parameters inside the input file.

By modifying the Makefile with the appropriate options it is very easy to obtain different versions of the application depending on the kind of infrastructure chosen for the analysis: standalone Linux, MPI or Grid-enabled versions.

Starting from the developed application different tests have been performed in order to validate results and compare performances obtained on different infrastructures. To this goal, tests have been divided into two different categories:

• Validation Tests

• Performance Tests

### Validation Tests

In order to validate the results obtained with the developed software, a small dataset coming from a published study [8] has been used for the analysis. The considered case study concerns the comparison of results obtained from separated analyses of HUVEC (Human Umbilical Vein Endothelial Cells) and Fibroblasts, derived from same donors, treated with Interferon-α (INF-α), to the purpose of identifying interferon's effects on transcriptome of endothelial cells.

The dataset is divided in two parts with the following features:

• ChipType: Affimetrix GeneChip HU-133A

• Origin: HUVEC, Human Umbilical Vein Endothelial Cells

• Genes: 22283

• Dataset dimension: 103 MB

• Number of microrarrays: 10

• ChipType: Affimetrix GeneChip HU-133A

• Origin: FB, Human Umbilical Fibroblasts

• Genes: 22283

• Dataset dimension: 54.3 MB

• Number of microrarrays: 5

The datasets are both composed by baseline and experiment arrays (respectively untreated and treated with INF-α) and for each of them the following steps, according to the original analysis, have been performed:

• Normalization: Invariant-set method

• Model-based expression: PM Only model [9,10]

• Extraction of differentially expressed genes: fold change with threshold 2.

As a first test, microarrays have been analyzed using both original and modified versions of dChip (standalone Linux version, parallel and grid-enabled). The same options have been set in all tests in order to compare final results.

**Figure 2:**
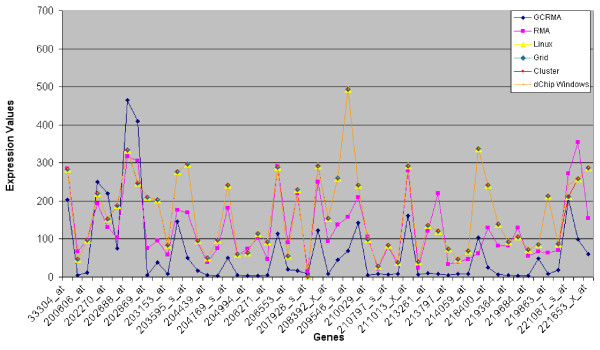
**Trend of (mean) expression values of baseline HUVEC arrays using R/Bioconductor and dChip algorithms**. A graphical representation of results presented on Table 1 and 3 is shown. It's worth noting that dChip versions results are overlapped and they have a similar trend compared to RMA algorithm.

**Figure 3:**
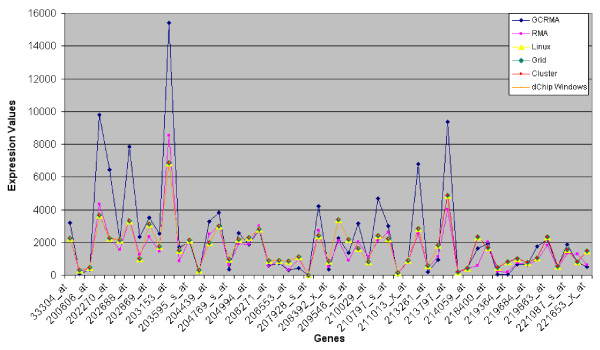
**Trend of (mean) expression values of experiment HUVEC arrays using R/Bioconductor and dChip algorithms**. A graphical representation of results presented on Table 2 and 4 is shown. It's worth noting that all dChip versions results are overlapped and they have a similar trend compared to RMA algorithm.

Tables 1 and 2 and Figures 2 and 3 represent the mean values of gene expressions, computed respectively on the baseline and experiment arrays on HUVEC data, coming from developed and original dChip versions. We notice that all the new dChip versions give the same results. There is a really small difference between Windows and Linux versions of dChip. This is probably due to the different approximations between compilers on Windows and Linux platforms. However, these little differences do not affect the global final result that can be considered pretty much the same.

**Table 1:** Comparison of baseline arrays results using all dChip implementations

**probe set**	**Gene Name**	**Linux**	**Cluster**	**Grid**	**Windows**
33304_at	interferon stimulated gene 20 kDa	284.20	284.20	284.20	284.02
823_at	chemokine (C-X3-C motif) ligand 1	47.16	47.16	47.16	47.09
200606_at	desmoplakin (DPI, DPII)	97.68	97.68	97.68	97.64
202269_x_at	guanylate binding protein 1	220.40	220.40	220.40	220.36
202270_at	guanylate binding protein 1	152.16	152.16	152.16	152.31
202687_s_at	tumor necrosis factor member 10	188.82	188.82	188.82	189.00
202688_at	tumor necrosis factor member 10	334.35	334.35	334.35	334.48
202748_at	guanylate binding prot 2, interf-inducible	247.40	247.40	247.40	247.66
202869_at	2',5'-oligoadenylate synthetase 1	209.99	209.99	209.99	209.87
203148_s_at	tripartite motif-containing 14	203.86	203.86	203.86	203.81
203153_at	interferon-induced protein	84.72	84.72	84.72	84.89
203236_s_at	lectin, galactoside-binding, soluble, 9	277.38	277.38	277.38	277.33
203595_s_at	retinoic acid- and interferon-inducible prot	296.66	296.66	296.66	296.56
204070_at	retinoic acid receptor responder 3	95.42	95.42	95.42	95.26
204439_at	chromosome 1 open reading frame 29	50.84	50.84	50.84	50.74
204533_at	chemokine (C-X-C motif) ligand 10	97.55	97.55	97.55	97.53
204769_s_at	transporter 2, ATP-binding cassette	241.98	241.98	241.98	241.99
204972_at	2'-5'-oligoadenylate synthetase 2	61.14	61.14	61.14	61.07
204994_at	myxovirus (influenza virus) resistance 2	63.91	63.91	63.91	63.85
205660_at	2'-5'-oligoadenylate synthetase-like	113.84	113.84	113.84	113.87
206271_at	toll-like receptor 3	92.93	92.93	92.93	92.72
206503_x_at	promyelocytic leukemia	288.58	288.58	288.58	288.80
206553_at	2'-5'-oligoadenylate synthetase 2	55.00	55.00	55.00	55.03
207375_s_at	interleukin 15 receptor, alpha	230.35	230.35	230.35	230.40
207928_s_at	glycine receptor, alpha 3	1.15	1.15	1.15	0.95
208012_x_at	SP110 nuclear body protein	291.32	291.32	291.32	290.87
208392_x_at	SP110 nuclear body protein	155.09	155.09	155.09	155.13
208436_s_at	interferon regulatory factor 7	259.61	259.61	259.61	259.68
209546_s_at	apolipoprotein L, 1	493.11	493.11	493.11	492.97
209969_s_at	signal transd and activator of transcription 1	241.42	241.42	241.42	241.65
210029_at	indoleamine-pyrrole 2,3 dioxygenase	99.88	99.88	99.88	99.70
210163_at	chemokine (C-X-C motif) ligand 11	27.88	27.88	27.88	27.81
210797_s_at	2'-5'-oligoadenylate synthetase-like	83.82	83.82	83.82	83.70
210846_x_at	tripartite motif-containing 14	38.65	38.65	38.65	38.51
211013_x_at	promyelocytic leukemia	292.75	292.75	292.75	292.74
211122_s_at	chemokine (C-X-C motif) ligand 11	40.68	40.68	40.68	40.83
213261_at	KIAA0342 gene product	135.93	135.93	135.93	135.74
213716_s_at	secreted and transmembrane 1	120.94	120.94	120.94	120.72
213797_at	vipirin	74.26	74.26	74.26	74.25
214038_at	chemokine (C-C motif) ligand 8	47.63	47.63	47.63	47.56
214059_at	interferon-induced protein 44	68.26	68.26	68.26	68.22
214329_x_at	ESTs	338.08	338.08	338.08	338.22
218400_at	2'-5'-oligoadenylate synthetase 3	242.05	242.05	242.05	242.01
219011_at	pleckstrin homology domain	139.71	139.71	139.71	139.58
219364_at	likely ortholog of mouse D11lgp2	92.46	92.46	92.46	92.17
219593_at	peptide transporter 3	105.99	105.99	105.99	106.00
219684_at	28 kD interferon responsive protein	71.72	71.72	71.72	71.69
219691_at	hypothetical protein FLJ20073	85.39	85.39	85.39	85.33
219863_at	cyclin-E binding protein 1	212.87	212.87	212.87	212.73
220104_at	likely ortholog of rat zinc-finger antivir prot	86.64	86.64	86.64	86.58
221087_s_at	apolipoprotein L, 3	211.18	211.18	211.18	242.92
221371_at	tumor necrosis factor member 18	258.60	258.60	258.60	258.67
221653_x_at	apolipoprotein L, 2	286.52	286.52	286.52	286.22

**Table 2:** Comparison of experiment arrays results using all dChip implementations

**probe set**	**Gene Name**	**Linux**	**Cluster**	**Grid**	**Windows**
33304_at	interferon stimulated gene 20 kDa	2244.54	2244.54	2244.54	2246.75
823_at	chemokine (C-X3-C motif) ligand 1	303.39	303.39	303.39	303.30
200606_at	desmoplakin (DPI, DPII)	459.07	459.07	459.07	459.40
202269_x_at	guanylate binding protein 1	3651.44	3651.44	3651.44	3654.06
202270_at	guanylate binding protein 1	2282.05	2282.05	2282.05	2283.43
202687_s_at	tumor necrosis factor member 10	2133.03	2133.03	2133.03	2131.72
202688_at	tumor necrosis factor member 10	3309.36	3309.36	3309.36	3311.57
202748_at	guanylate binding prot 2, interf-inducible	1007.71	1007.71	1007.71	1007.97
202869_at	2',5'-oligoadenylate synthetase 1	3112.07	3112.07	3112.07	3112.08
203148_s_at	tripartite motif-containing 14	1774.94	1774.94	1774.94	1773.47
203153_at	interferon-induced protein	6863.81	6863.81	6863.81	6853.83
203236_s_at	lectin, galactoside-binding, soluble, 9	1540.56	1540.56	1540.56	1540.34
203595_s_at	retinoic acid- and interfer-inducible prot	2144.76	2144.76	2144.76	2144.24
204070_at	retinoic acid receptor responder 3	314.03	314.03	314.03	314.49
204439_at	chromosome 1 open reading frame 29	1982.76	1982.76	1982.76	1980.53
204533_at	chemokine (C-X-C motif) ligand 10	3018.77	3018.77	3018.77	3015.67
204769_s_at	transporter 2, ATP-binding cassette	971.37	971.37	971.37	973.42
204972_at	2'-5'-oligoadenylate synthetase 2	2173.07	2173.07	2173.07	2172.05
204994_at	myxovirus (influenza virus) resistance 2	2312.15	2312.15	2312.15	2312.96
205660_at	2'-5'-oligoadenylate synthetase-like	2793.93	2793.93	2793.93	2792.91
206271_at	toll-like receptor 3	905.37	905.37	905.37	905.16
206503_x_at	promyelocytic leukemia	910.07	910.07	910.07	909.93
206553_at	2'-5'-oligoadenylate synthetase 2	873.65	873.65	873.65	873.49
207375_s_at	interleukin 15 receptor, alpha	1114.76	1114.76	1114.76	1114.91
207928_s_at	glycine receptor, alpha 3	2.37	2.37	2.37	2.65
208012_x_at	SP110 nuclear body protein	2436.07	2436.07	2436.07	2435.94
208392_x_at	SP110 nuclear body protein	842.89	842.89	842.89	842.75
208436_s_at	interferon regulatory factor 7	3397.64	3397.64	3397.64	3397.97
209546_s_at	apolipoprotein L, 1	2170.99	2170.99	2170.99	2170.16
209969_s_at	signal transd and activator of transcription 1	1642.44	1642.44	1642.44	1642.01
210029_at	indoleamine-pyrrole 2,3 dioxygenase	814.88	814.88	814.88	814.71
210163_at	chemokine (C-X-C motif) ligand 11	2400.30	2400.30	2400.30	2400.22
210797_s_at	2'-5'-oligoadenylate synthetase-like	2210.17	2210.17	2210.17	2210.97
210846_x_at	tripartite motif-containing 14	139.55	139.55	139.55	139.69
211013_x_at	promyelocytic leukemia	891.32	891.32	891.32	892.07
211122_s_at	chemokine (C-X-C motif) ligand 11	2835.46	2835.46	2835.46	2829.05
213261_at	KIAA0342 gene product	598.16	598.16	598.16	598.27
213716_s_at	secreted and transmembrane 1	1844.02	1844.02	1844.02	1846.77
213797_at	vipirin	4883.14	4883.14	4883.14	4882.36
214038_at	chemokine (C-C motif) ligand 8	209.79	209.79	209.79	209.81
214059_at	interferon-induced protein 44	417.94	417.94	417.94	418.01
214329_x_at	ESTs	2344.63	2344.63	2344.63	2348.62
218400_at	2'-5'-oligoadenylate synthetase 3	1695.06	1695.06	1695.06	1694.47
219011_at	pleckstrin homology domain	462.15	462.15	462.15	462.03
219364_at	likely ortholog of mouse D11lgp2	828.68	828.68	828.68	830.36
219593_at	peptide transporter 3	1005.54	1005.54	1005.54	1006.65
219684_at	28 kD interferon responsive protein	792.34	792.34	792.34	793.37
219691_at	hypothetical protein FLJ20073	1068.37	1068.37	1068.37	1068.61
219863_at	cyclin-E binding protein 1	2329.30	2329.30	2329.30	2332.30
220104_at	likely ortholog of rat zinc-finger antivir prot	547.91	547.91	547.91	548.08
221087_s_at	apolipoprotein L, 3	1547.18	1547.18	1547.18	1570.80
221371_at	tumor necrosis factor member 18	821.30	821.30	821.30	820.92
221653_x_at	apolipoprotein L, 2	1495.00	1495.00	1495.00	1496.70

As a second test, the same analysis has been performed by using R/Bioconductor software using both GCRMA [11] and RMA [12] algorithms and results have been compared with the previous obtained with dChip.

This comparison is principally for completeness purposes since the dataset was published with results coming from an R/Bioconductor analysis.

Although there are currently many different methods for processing and summarizing probe level data from Affymetrix oligonucleotide arrays, R/Bioconductor and dChip are two of the most popular methods that consistently produce the best agreement between oligo array and RT-PCR data for medium and high intensity genes [13,14]. It is known that often expression values computed with dChip and RMA algorithms show similar results, while results are different for GCRMA.

Tables 3 and 4 and Figures 2 and 3 show the comparison between dChip Linux and R/Bioconductor results obtained on the former data. It's observable that dChip and RMA present similar trends conversely to GCRMA results.

**Table 3:** Comparison of baseline arrays results using R/Bioconductor and dChip implementation

**probe set**	**Gene Name**	**GCRMA**	**RMA**	**dChip Linux**
33304_at	interferon stimulated gene 20 kDa	203.01	284.72	284.20
823_at	chemokine (C-X3-C motif) ligand 1	4.93	66.60	47.16
200606_at	desmoplakin (DPI, DPII)	12.38	97.36	97.68
202269_x_at	guanylate binding protein 1	250.15	192.46	220.40
202270_at	guanylate binding protein 1	220.16	131.76	152.16
202687_s_at	tumor necrosis factor member 10	75.06	104.00	188.82
202688_at	tumor necrosis factor member 10	464.46	316.93	334.35
202748_at	guanylate binding protein 2, interferon-inducible	409.70	305.82	247.40
202869_at	2',5'-oligoadenylate synthetase 1	5.81	75.13	209.99
203148_s_at	tripartite motif-containing 14	38.57	96.29	203.86
203153_at	interferon-induced protein	7.75	58.02	84.72
203236_s_at	lectin, galactoside-binding, soluble, 9	146.27	176.17	277.38
203595_s_at	retinoic acid- and interferon-inducible protein	50.34	169.34	296.66
204070_at	retinoic acid receptor responder 3	16.93	90.63	95.42
204439_at	chromosome 1 open reading frame 29	5.06	39.15	50.84
204533_at	chemokine (C-X-C motif) ligand 10	4.16	76.22	97.55
204769_s_at	transporter 2, ATP-binding cassette	50.42	180.53	241.98
204972_at	2'-5'-oligoadenylate synthetase 2	4.97	56.39	61.14
204994_at	myxovirus (influenza virus) resistance 2	3.85	74.62	63.91
205660_at	2'-5'-oligoadenylate synthetase-like	3.64	101.76	113.84
206271_at	toll-like receptor 3	5.82	46.99	92.93
206503_x_at	promyelocytic leukemia	114.42	292.54	288.58
206553_at	2'-5'-oligoadenylate synthetase 2	19.89	90.71	55.00
207375_s_at	interleukin 15 receptor, alpha	17.48	219.39	230.35
207928_s_at	glycine receptor, alpha 3	8.48	14.27	1.15
208012_x_at	SP110 nuclear body protein	122.80	250.06	291.32
208392_x_at	SP110 nuclear body protein	8.20	94.29	155.09
208436_s_at	interferon regulatory factor 7	45.15	138.04	259.61
209546_s_at	apolipoprotein L, 1	69.01	158.61	493.11
209969_s_at	signal transducer and activator of transcription 1	142.29	210.42	241.42
210029_at	indoleamine-pyrrole 2,3 dioxygenase	5.07	105.59	99.88
210163_at	chemokine (C-X-C motif) ligand 11	7.94	22.10	27.88
210797_s_at	2'-5'-oligoadenylate synthetase-like	6.44	80.66	83.82
210846_x_at	tripartite motif-containing 14	9.07	34.13	38.65
211013_x_at	promyelocytic leukemia	161.42	279.82	292.75
211122_s_at	chemokine (C-X-C motif) ligand 11	7.12	23.88	40.68
213261_at	KIAA0342 gene product	10.03	120.40	135.93
213716_s_at	secreted and transmembrane 1	8.37	220.23	120.94
213797_at	vipirin	4.70	33.70	74.26
214038_at	chemokine (C-C motif) ligand 8	8.16	39.84	47.63
214059_at	interferon-induced protein 44	7.65	46.42	68.26
214329_x_at	ESTs	104.51	62.56	338.08
218400_at	2'-5'-oligoadenylate synthetase 3	25.02	128.86	242.05
219011_at	pleckstrin homology domain	5.92	81.93	139.71
219364_at	likely ortholog of mouse D11lgp2	4.44	81.92	92.46
219593_at	peptide transporter 3	3.52	129.28	105.99
219684_at	28 kD interferon responsive protein	3.66	55.44	71.72
219691_at	hypothetical protein FLJ20073	48.86	66.73	85.39
219863_at	cyclin-E binding protein 1	8.03	64.18	212.87
220104_at	likely ortholog of rat zinc-finger antiviral protein	18.28	70.07	86.64
221087_s_at	apolipoprotein L, 3	204.63	272.26	211.18
221371_at	tumor necrosis factor member 18	98.32	354.34	258.60
221653_x_at	apolipoprotein L, 2	60.81	153.69	286.52

**Table 4:** Comparison of experiment arrays results using R/Bioconductor and dChip implementation

**probe set**	**Gene Name**	**GCRMA**	**RMA**	**dChip Linux**
33304_at	interferon stimulated gene 20 kDa	3196.51	2268.74	2244.54
823_at	chemokine (C-X3-C motif) ligand 1	94.32	199.31	303.39
200606_at	desmoplakin (DPI, DPII)	346.10	472.57	459.07
202269_x_at	guanylate binding protein 1	9812.82	4339.89	3651.44
202270_at	guanylate binding protein 1	6437.35	2278.30	2282.05
202687_s_at	tumor necrosis factor member 10	2156.88	1550.21	2133.03
202688_at	tumor necrosis factor member 10	7854.88	3398.70	3309.36
202748_at	guanylate binding protein 2, interferon-inducible	2345.96	1250.53	1007.71
202869_at	2',5'-oligoadenylate synthetase 1	3531.07	2325.40	3112.07
203148_s_at	tripartite motif-containing 14	2521.04	1435.02	1774.94
203153_at	interferon-induced protein	15412.19	8556.41	6863.81
203236_s_at	lectin, galactoside-binding, soluble, 9	1711.84	847.55	1540.56
203595_s_at	retinoic acid- and interferon-inducible protein	2012.55	2181.61	2144.76
204070_at	retinoic acid receptor responder 3	211.37	292.39	314.03
204439_at	chromosome 1 open reading frame 29	3293.52	2515.21	1982.76
204533_at	chemokine (C-X-C motif) ligand 10	3827.84	3009.14	3018.77
204769_s_at	transporter 2, ATP-binding cassette	351.22	608.05	971.37
204972_at	2'-5'-oligoadenylate synthetase 2	2566.06	1918.87	2173.07
204994_at	myxovirus (influenza virus) resistance 2	1879.30	1864.21	2312.15
205660_at	2'-5'-oligoadenylate synthetase-like	2757.19	3007.87	2793.93
206271_at	toll-like receptor 3	572.08	560.92	905.37
206503_x_at	promyelocytic leukemia	737.59	913.65	910.07
206553_at	2'-5'-oligoadenylate synthetase 2	315.50	323.83	873.65
207375_s_at	interleukin 15 receptor, alpha	438.00	1020.22	1114.76
207928_s_at	glycine receptor, alpha 3	8.43	14.61	2.37
208012_x_at	SP110 nuclear body protein	4220.13	2720.23	2436.07
208392_x_at	SP110 nuclear body protein	347.25	510.43	842.89
208436_s_at	interferon regulatory factor 7	2270.19	2097.07	3397.64
209546_s_at	apolipoprotein L, 1	1379.27	915.53	2170.99
209969_s_at	signal transducer and activator of transcription 1	3157.23	2027.40	1642.44
210029_at	indoleamine-pyrrole 2,3 dioxygenase	833.17	1133.97	814.88
210163_at	chemokine (C-X-C motif) ligand 11	4702.03	2094.38	2400.30
210797_s_at	2'-5'-oligoadenylate synthetase-like	3022.68	2622.26	2210.17
210846_x_at	tripartite motif-containing 14	20.39	70.48	139.55
211013_x_at	promyelocytic leukemia	952.73	861.97	891.32
211122_s_at	chemokine (C-X-C motif) ligand 11	6782.66	2521.40	2835.46
213261_at	KIAA0342 gene product	184.92	481.56	598.16
213716_s_at	secreted and transmembrane 1	924.99	1132.93	1844.02
213797_at	vipirin	9384.39	4037.25	4883.14
214038_at	chemokine (C-C motif) ligand 8	131.65	153.26	209.79
214059_at	interferon-induced protein 44	460.99	417.83	417.94
214329_x_at	ESTs	1651.68	566.67	2344.63
218400_at	2'-5'-oligoadenylate synthetase 3	1914.27	2042.07	1695.06
219011_at	pleckstrin homology domain	33.03	216.27	462.15
219364_at	likely ortholog of mouse D11lgp2	23.39	211.10	828.68
219593_at	peptide transporter 3	678.07	754.28	1005.54
219684_at	28 kD interferon responsive protein	678.29	651.96	792.34
219691_at	hypothetical protein FLJ20073	1761.43	914.96	1068.37
219863_at	cyclin-E binding protein 1	2191.56	1844.42	2329.30
220104_at	likely ortholog of rat zinc-finger antiviral protein	500.61	427.62	547.91
221087_s_at	apolipoprotein L, 3	1873.08	1325.73	1547.18
221371_at	tumor necrosis factor member 18	787.94	1269.50	821.30
221653_x_at	apolipoprotein L, 2	520.21	609.52	1495.00

Ultimately, results of the entire analysis related to differences between HUVEC and FB are illustrated. We have found that using all dChip developed versions, in HUVEC, 239 genes were up-regulated (> 2-fold increase) by IFN, including genes involved in the host response to RNA viruses, inflammation, and apoptosis. Interestingly, 35 genes showed a > 4-fold higher induction compared with human fibroblasts. Obviously, because the results of the published study had been obtained using GCRMA algorithm, they are not exactly the same of dChip's. These show 175 genes up-regulated by IFNs in HUVEC and 41 genes with a > 5-fold higher induction compared with human fibroblasts. However it's interesting to notice that quite similar results have been found.

In particular (Table 5) we have found that CXCL11 (chemokine (C-X-C motif) ligand 11) is selectively induced by IFN-α along with other genes associated with angiogenesis regulation, including CXCL10, TRAIL, and guanylate-binding protein 1.

**Table 5:** induction of IFN-α on HUVEC respect to Human Fibroblasts

**probe set**	**Gene Name**	**HUVEC/FB fold change increment**
33304_at	interferon stimulated gene 20 kDa	3.61
823_at	chemokine (C-X3-C motif) ligand 1	8.88
200606_at	desmoplakin (DPI, DPII)	4
***202269_x_at***	***guanylate binding protein 1, interferon-inducible***	***3.17***
***202270_at***	***guanylate binding protein 1, interferon-inducible***	***3.79***
***202687_s_at***	***tumor necrosis factor (ligand) superfamily, member 10***	***5.37***
***202688_at***	***tumor necrosis factor (ligand) superfamily, member 10***	***12.49***
***202748_at***	***guanylate binding protein 2, interferon-inducible***	***4.55***
202869_at	2',5'-oligoadenylate synthetase 1, 40/46 kDa	6.69
203148_s_at	tripartite motif-containing 14	3.34
203153_at	interferon-induced protein with tetratricopeptide repeats 1	3.18
203236_s_at	lectin, galactoside-binding, soluble, 9 (galectin 9)	6.49
203595_s_at	retinoic acid- and interferon-inducible protein (58 kD)	3.67
204070_at	retinoic acid receptor responder (tazarotene induced) 3	5.09
204439_at	chromosome 1 open reading frame 29	5.53
***204533_at***	***chemokine (C-X-C motif) ligand 10***	***16.73***
204769_s_at	transporter 2, ATP-binding cassette, sub-family B (MDR/TAP)	5.04
204972_at	2'-5'-oligoadenylate synthetase 2, 69/71 kDa	12.99
204994_at	myxovirus (influenza virus) resistance 2 (mouse)	3.71
205660_at	2'-5'-oligoadenylate synthetase-like	3.5
206271_at	toll-like receptor 3	3.06
206503_x_at	promyelocytic leukemia	3.38
206553_at	2'-5'-oligoadenylate synthetase 2, 69/71 kDa	9.08
207375_s_at	interleukin 15 receptor, alpha	4.1
207928_s_at	glycine receptor, alpha 3	4.52
208012_x_at	SP110 nuclear body protein	3.11
208392_x_at	SP110 nuclear body protein	5.67
208436_s_at	interferon regulatory factor 7	4.09
209546_s_at	apolipoprotein L, 1	4.15
209969_s_at	signal transducer and activator of transcription 1, 91 kDa	4.17
210029_at	indoleamine-pyrrole 2,3 dioxygenase	8
***210163_at***	***chemokine (C-X-C motif) ligand 11***	***76.55***
210797_s_at	2'-5'-oligoadenylate synthetase-like	12.49
210846_x_at	tripartite motif-containing 14	4.91
211013_x_at	promyelocytic leukemia	3.5
***211122_s_at***	***chemokine (C-X-C motif) ligand 11***	***19.03***
213261_at	KIAA0342 gene product	3.97
213716_s_at	secreted and transmembrane 1	7.2
213797_at	Vipirin	5.06
214038_at	chemokine (C-C motif) ligand 8	3.57
214059_at	interferon-induced protein 44	4.58
214329_x_at	ESTs, Weakly similar to cytokine receptor-like factor 2	3.88
218400_at	2'-5'-oligoadenylate synthetase 3, 100 kDa	4.6
219011_at	pleckstrin homology domain containing, family A member 4	4.28
219364_at	likely ortholog of mouse D11lgp2	3.92
219593_at	peptide transporter 3	4.54
219684_at	28 kD interferon responsive protein	6.4
219691_at	hypothetical protein FLJ20073	4.46
219863_at	cyclin-E binding protein 1	7.68
220104_at	likely ortholog of rat zinc-finger antiviral protein	5.39
221087_s_at	apolipoprotein L, 3	5.47
***221371_at***	***tumor necrosis factor (ligand) superfamily, member 18***	***3.67***
221653_x_at	apolipoprotein L, 2	3.77

The induction of IFN-α on HUVEC respect to Human Fibroblasts is shown. In particular the up-regulated HUVEC genes with fold induction more then 3 higher compared with Fibroblast's are shown. The genes associated with angiogenesis induction are marked with bold text. As we expected, results show that CXCL11 gene is the most discriminator.

### Performance Tests

These tests, although far from any biological meaning, have the only purpose of comparing performances using a large dataset on different environments. In details several application tests have been performed on both cluster and Grid environments using different values of parallelization rate and final results have been compared.

In order to create a large data set for testing purposes, the on-line public repository GEO [15] has been used. A dataset of 1000 HG-U133A Breast Cancer microarrays has been made available. It shows the following features:

• ChipType: Affimetrix GeneChip HU-133A

• Origin: Breast Cancer

• Genes: 22283

• Dataset dimension: ~12 GB

• Number of microrarray: 1000

The performance results have been obtained by calculating the average of the execution times of three independent executions of the application on the same dataset.

The tests on the cluster environment have been performed using the MPI implementation of dChip on Michelangelo, the LITBIO project cluster [16] dedicated to bioinformatics applications requiring great computational efforts.

Grid tests have been performed using the gLite middleware on the BIOMED Virtual Organization [17] of the EGEE (Enabling Grids for E-sciencE) infrastructure [18]. In this case data had been previously uploaded on several remote and distributed storage elements and have been analyzed submitting more parallel jobs through opportune strategies.

Concerning Grid tests, a not-MPI parallel implementation has been used, because MPI jobs are unstable on the gLite middleware, at the present time. More parallel jobs can be submitted and monitored using ad hoc scripts.

Two kinds of test have been carried out: (i) scalability on the number of parallel jobs, (ii) scalability on the number of microarrays.

**Figure 4:**
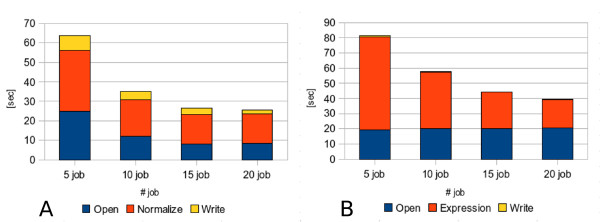
**Scalability on cluster environment by increasing parallelization rate**. The different scalability of dChip MPI version run on LITBIO cluster using 100 microarrays respectively for (A) module 1 (normalization), (B) module2 (model-based expression computation) is shown. In particular the mean execution times coming from three independent executions of respectively 5, 10, 15 and 20 parallel jobs are represented.

**Figure 5:**
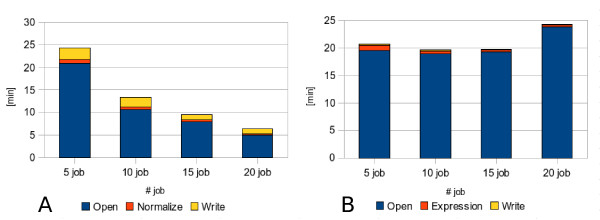
**Scalability on Grid environment by increasing parallelization rate**. The different scalability of dChip Grid version run within BIOMED Virtual Organization using 100 microarrays respectively for (A) module 1 (normalization), (B) module2 (model-based expression computation) is shown. In particular the mean execution times coming from three independent executions of respectively 5, 10, 15 and 20 parallel jobs are represented.

As a first test, a subset of 100 microarrays has been analyzed to compare the performances of the two parallel modules in changing the parallelization rate, both on cluster and grid implementation. In detail, four tests have been run using respectively 5, 10, 15 and 20 parallel jobs. By comparing results, we can observe that Module 1 has a better scalability (Figures 4A and 5A) due to the different parallelization strategy adopted. In fact, while in the first module there is a reduction in time for all the three execution steps (file opening, normalization and output writing), in the Module 2 (Figures 4B and 5B) we have a reduction only for gene expression calculation whereas file opening and output writing remain constant. Through the comparison of cluster and Grid executions we can notice that this trend is approximately the same in both conditions. Eventually, the speedup ratio (S(N) = T(1)/T(N), where T(1) is the execution time on a single processor and T(N) the execution time on N processors), has been calculated for the cluster tests with the purpose to estimate the parallelization efficiency. As shown in Table 6, it is worth noting that for 100 microarrays the parallelization has a quite good result up to 15 parallel jobs.

**Table 6:** Mean execution times and speedup ratio analysing 100 microarrays on cluster environment

	**1 job**	**5 job**	**10 job**	**15 job**	**20 job**
**Module 1**	285 sec	63.80 sec	35.20 sec	26.40 sec	25.65 sec
**Module 2**	344 sec	81.60 sec	58.00 sec	44.47 sec	39.55 sec
**Module 1+2**	629 sec	145.40 sec	93.20 sec	70.87 sec	65.20 sec
					
**speedup ratio module 1**		4.47	8.09	10.79	11.11
**speedup ratio module 2**		4.22	5.93	7.74	8.69
**speedup ratio module 1+2**		4.33	6.75	8.88	9.65

The table shows the mean execution times coming from three different execution of dChip MPI version run on LITBIO cluster using respectively 1, 5, 10, 15, 20 parallel jobs to compute the expression values of 100 microarrays. To estimate the parallelization efficiency, the speedup ratio (S(N) = T(1)/T(N), where T(1) is the execution time on a single processor and T(N) the execution time on N processors) was calculated.

**Figure 6:**
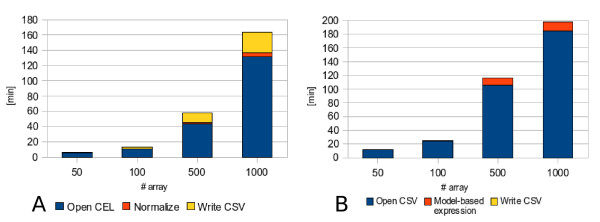
**Scalability on Grid environment by increasing microarrays' number**. The different scalability on microarray's number of dChip Grid version run within BIOMED Virtual Organization using 10 parallel jobs respectively for (A) module 1 (normalization), (B) module2 (model-based expression computation) is shown. In particular the mean execution times coming from three independent executions to analyse respectively 50, 100, 500 and 1000 microarrays are represented.

As a second test, the whole 1000 microarrays dataset has been analyzed on the Grid, by running the parallel dChip version using 10 parallel jobs, in order to investigate the trend of performances according to the dataset dimensions. In Figure 6 we can see the results concerning respectively the Module 1 and Module 2 executions. Cluster performances show better results than the Grid ones.

Actually the great advantage for researchers in using Grid is the possibility to store a large amount of data, to run complex algorithms and to access all data shared by Grid virtual communities, using remote resources.

This feature has a great relevance especially for small laboratories or for researchers that, due to the high cost of producing microarray data, cannot perform analyses using large datasets (a necessary condition to get better results). The access to a Grid environment makes it possible to get access to all the datasets made available by the community and to perform more accurate analyses.

As previously said, the Grid provides several advantages related to security aspects as well. Using the Grid certificate-based authentication data are safe from possible attacks to privacy and security.

Finally, the integration of this application into a Grid-enabled portal provides a simple graphical user interface to run the application. In this way, researchers do not need any particular hardware or software installed locally but only a web connection to the portal.

Actually, besides the cost of producing data, another relevant issue concerns the analysis of large datasets using standalone applications running on local hardware. The use of such applications implies the existence powerful computers available locally, and, often, this is not possible, even in large laboratories. The previously explained analysis related to 1000 microarrays is an example of experiment that could not be performed using standalone applications, even on the most recent powerful computers.

Our solution resolves this problem and provides users with a web-based service able to launch more analyses in a parallel way, easily monitoring the status of executions directly from the portal.

## Conclusion

A scalable way to analyze large microarray datasets has been presented. To do that, we have ported existing tools to High Performance and Grid Computing environments. dChip software has been ported on Linux platforms and modified, by using appropriate parallelization strategies, to permit its execution on both cluster environments and Grid infrastructures. The added value provided by the use of Grid technologies is the possibility of exploiting both computational and data Grids infrastructures to analyze large datasets of distributed data. The software has been successfully validated through the comparison with the original standalone Windows version of dChip. Performance tests were performed in order to investigate the improvements on performances related to the adopted strategies for parallelization. Moreover these tests have been used to compare cluster and Grid performances too. As result we found that parallelization gives quite good results in terms of execution times, especially for the first module. Furthermore we found that Grid executions have longer execution times rather than cluster ones. But it is worth noting that the relevance related to the use of Grid computing for the presented application is principally focused on the opportunity of sharing data. This is done through different research groups and exploiting distributed computational resources rather than on the improvement of performances.

## Availability and requirements

• **Project name: **grid-dChip

• **Project home page: **

A web-portal version of the software is accessible within the LITBIO portal.

• **Operating system(s): **Linux

• **Programming language: **C++

• **Other requirements: **MPICH2 library (cluster version)

• **License: **GNU GPL

## Abbreviations

GFAL: Grid File Access Library; API: Application Program Interface; INF: Interferon; HUVEC: Human Umbilical Vein Endothelial Cells; FB: Human Fibroblasts

## Authors' contributions

LC developed the code, participated in the software design and tests definition, ran validation and performance tests and helped to draft the manuscript, MF helped to draft the manuscript, IP participated in the software design, helped to define the performance tests and to draft the manuscript, SS helped to define the validation tests and to draft the manuscript, LT participated in the Grid-design of the software, helped to define the performance tests and to draft the manuscript. All authors read and approved the final manuscript.

## Supplementary Material

Additional file 1 **Source code.** The archive contains the source code of modified dChip. Click here for file
